# Developing Local Board of Health Guidelines to Promote Healthy Food Access — King County, Washington, 2010–2012

**DOI:** 10.5888/pcd12.140544

**Published:** 2015-04-30

**Authors:** Emilee Quinn, Donna B. Johnson, James Krieger, Erin MacDougall, Elizabeth Payne, Nadine L. Chan

**Affiliations:** Author Affiliations: Donna B. Johnson, Elizabeth Payne, University of Washington, Seattle, Washington; James Krieger, University of Washington, Seattle, Washington, and Action for Healthy Food, Seattle, Washington; Erin MacDougall, Bastyr University, Kenmore, Washington; Nadine L. Chan, Public Health – Seattle and King County, Seattle, Washington. During this study, James Krieger and Erin MacDougall were affiliated with Public Health – Seattle and King County, Seattle, Washington.

## Abstract

Policies that change environments are important tools for preventing chronic diseases, including obesity. Boards of health often have authority to adopt such policies, but few do so. This study assesses 1) how one local board of health developed a policy approach for healthy food access through vending machine guidelines (rather than regulations) and 2) the impact of the approach. Using a case study design guided by “three streams” policy theory and RE-AIM, we analyzed data from a focus group, interviews, and policy documents. The guidelines effectively supported institutional policy development in several settings. Recognition of the problem of chronic disease and the policy solution of vending machine guidelines created an opening for the board to influence nutrition environments. Institutions identified a need for support in adopting vending machine policies. Communities could benefit from the study board’s approach to using nonregulatory evidence-based guidelines as a policy tool.

## Background

Experts increasingly call for policies to prevent chronic diseases, including obesity. Such policies aim to improve access to healthy food and physical activity opportunities, making it easier for the population to adopt healthier behaviors (1,2). Local boards of health (LBOH) often have authority to adopt policies that could influence institutions such as government offices and worksites, housing, and recreational facilities (3,4).

Most local health departments engage in policy-making activities (eg, preparing issue briefs, providing public testimony) (5). Nearly half report policy making specific to obesity or chronic disease (5). However, fewer LBOHs engage in policy making than are authorized to do so (6). The form and policy-making authority of the approximately 3,200 LBOHs in the United States vary. More LBOHs are allowed to adopt regulations than to impose taxes, for example (5).

Public health practitioners could benefit from studies that deepen understanding of the feasibility, adoption, and implementation of policy approaches (7–10). Well respected theoretical frameworks (11,12) can inform such studies. This article examines the case of an innovative LBOH policy tool in King County, Washington, in the form of nonregulatory nutritional guidelines for food and beverages sold in vending machines, and we provide insight into the tool’s development and initial use.

## Methods

Using a qualitative case study design, this study assesses how one LBOH developed a policy approach to address healthy food access in its community and the extent of the approach’s initial use. The evaluation was designed through collaboration between public health practitioners and researchers affiliated with Washington’s Nutrition and Obesity Policy Research and Evaluation Network (NOPREN), a project funded by the Centers for Disease Control and Prevention to support transdisciplinary policy research and evaluation across a continuum of policy identification, development, evaluation, and dissemination (http://www.hsph.harvard.edu/nopren). Data were collected through focus group, interview, and document review methods.

We used Kingdon’s “three streams” theory (11) to describe the adoption of the King County Healthy Vending Guidelines (*Guidelines*) by the King County Board of Health (KCBOH). Kingdon’s constructs of “problem,” “policy,” and “political” streams are applied to consider whether and how local factors created a “policy window” for passage. We used the RE-AIM framework for policy impact (12) to guide an analysis of the use of the *Guidelines* by local jurisdictions (eg, cities) and organizations in the year following the adoption (Table 1).

**Table 1:** Theoretical Models and Constructs Used for the Study of King County Board of Health Healthy Vending Guidelines

Theory or Framework	Construct	Description
Policy development: “three streams” theory (11)	Problems stream	“Problems are brought to the attention of people in and around government by systematic indicators, by focusing events like crises and disasters, or by feedback from the operation of current programs” (p. 19).
		“How do conditions come to be defined as problems? Values, comparisons, and categories contribute to the translation” (p. 110).
	Policy stream	“The proposals that survive to the status of serious consideration meet several criteria, including their technical feasibility, their fit with dominant values and the current national mood, their budgetary workability, and the political support or opposition they might experience” (pp. 10–20).
	Political stream	“Flowing along independently of the problems and policy streams is the political stream, composed of such things as public mood, pressure group campaigns, election results, partisan or ideological districts in Congress, and changes of administration” (p. 145).
		The “mood-elections” combination . . . can force some subjects high on the agenda, and can also make it virtually impossible for government to pay serious attention to others. But once the item is on the agenda, the organized forces enter the picture, trying as best they can to bend the outcomes to their advantage ” (p.164).
	Policy window	“The separate streams of problems, policies, and politics come together at certain critical times. Solutions become joined to problems, and both of them are joined to favorable political forces” (p. 194).
Policy impact: adapted “RE-AIM” Framework (12)	Reach	“[T]he absolute number, percentage, and representativeness of those affected by the policy, or those whose health is to be improved as a result of the policy” (p. 108).
	Effectiveness	“[T]he change in proximal, or temporally appropriate, outcomes and any adverse impacts” (p. 108).
	Adoption	“[T]he absolute number, percentage, and representativeness of organizations, institutions, or governing bodies that pass or decide to implement a policy [and allocate] resources for enforcement, if applicable” (p. 108).
	Implementation^a^	“[A]pplying the policy as planned, adequately enforcing it, and ensuring ongoing and consistent compliance with the core components of the policy” (p. 109).
	Maintenance^a^	“[C]ompliance with the policy and resulting individual behavior changes and health outcomes that occur over time” and “continued enforcement of and compliance with the policy over time” (p. 109).

a Implementation and Maintenance are not addressed in this study, given the focus on preliminary impact within the first year of *Guidelines’* use.

Data included transcripts from a 1.5-hour focus group (October 2011) with all 4 local health department staff who participated most in guideline development, interviews with 4 LBOH members (February–August 2012; 3 local elected officials, 1 health expert), and interviews with representatives of 5 local jurisdictions and organizations that used the *Guidelines* (April–May 2012; 2 municipal staff members, 2 department directors, and 1 contract staff member). Nine women and 4 men participated. Interviews took 30 to 60 minutes. All but 2 were audio recorded; the interviewer took notes for each. Participants provided consent per protocol approved by University of Washington’s Human Subjects Division. We also reviewed meeting minutes and videos, policy drafts, memos, and contract language related to the LBOH’s adoption of the *Guidelines* and local jurisdictions’ vending machine policy development.

We developed code definitions based on the theoretical frameworks and research questions, and used Atlas.ti version 7.0 (ATLAS.ti GmbH) to code and analyze data. Two researchers coded the data, compared their coding, and resolved discrepancies as needed. One researcher reviewed and summarized the final coded passages. This same researcher reviewed policy documents and media reports to supplement data recordings from interviews, focus groups, and meetings. Two local health department staff reviewed the resulting narrative to vet data interpretation. Minor adjustments were made on the basis of feedback.

## Results

### Guidelines’ development

KCBOH is charged to “set county-wide public health policy, enact and enforce local public health regulations, and carry out other duties of local boards of health specified in state law” (13). Membership comprises 8 elected officials from specified jurisdictions (eg, county, largest city, 2 suburban cities) and 3 appointed health experts. KCBOH and its corresponding local health department, Public Health Seattle-King County (PHSKC), cover a large urban county with 39 cities, including Seattle, the most populous city in the state.

### Problems stream

Addressing obesity was a high priority for PHSKC and KCBOH. Public health surveillance indicated that obesity was a problem in King County. “The board of health every year tries to look at how we’re doing in King County,” said one KCBOH member. “It became very clear that we had a rising problem of obesity.” Broad-based community-wide prevention initiatives, such as the Obesity Prevention Initiative (14) and Communities Putting Prevention to Work (CPPW), increased attention to these issues. For example, the Seattle-King County CPPW project was a $25.5 million federally funded initiative focused on preventing obesity and tobacco use through policy, systems, and environmental change from 2010 through 2012 (15). PHSKC and KCBOH were impressed with evidence that frequent eating outside the home contributed to obesity and therefore wanted to improve the quality of food in away-from-home settings. At the time, there was limited practical guidance for aligning these food environments with the *2010 Dietary Guidelines for Americans* (DGA) (16).

### Policy stream

In 2010, KCBOH convened a Healthy Eating and Active Living (HEAL) subcommittee to examine potential actions to promote HEAL and prevent obesity. The subcommittee comprised 3 KCBOH members who were elected officials, 1 KCBOH member who was a health expert, and PHSKC staff members. The subcommittee created a list of approximately 25 best-practice policy strategies and selected several to implement, including guidelines for healthy vending. Strategies were chosen because they could reach many people, used approaches grounded in public health science, avoided redundancy, demonstrated leadership, and allowed flexibility for jurisdictions and businesses.

Before 2011, KCBOH had 2 policy categories: 1) Rules and Regulations, which “have the force of law, are general and permanent in nature, and are codified” and 2) Resolutions, which are statements “in support of a current action or project” that are neither permanent nor have the force of law (17). In early 2011, at the suggestion of the PHSKC director, KCBOH adopted a third policy category: Guidelines and Recommendations. The new category was “designed to provide policy guidance where the Board does not have regulatory authority and to increase the reach of public health policy making to sectors that have not considered the public health impact of their policies in their sectors” (17). Members saw the category as allowing for greater specificity and impact than resolutions, without the legal and political complexity of a rule or regulation. A KCBOH member said, “We could take more frequent action without having the legal consequences which sometimes seem to stymie government.” KCBOH used the category to create guidelines for healthy community planning and then for healthy vending.

It took approximately 6 months to develop the guidelines. PHSKC staff reviewed vending and nutrition guidelines and spoke with nutrition policy experts from across the United States, vending machine company representatives, and entities experienced with healthy vending. The resulting *Guidelines* categorized foods as “limited” (ie, most processed; highest levels of sodium, sugar, fat, and salt), “healthier,” and “healthiest” (ie, least processed, nutrient-rich, no added sugar or salt) based on the DGA and recommended increasing the proportion of “healthier” and “healthiest” items in vending machines (18) (Figure). They also provided guidance on using the *Guidelines* for policy development. The subcommittee identified “early adopter” organizations to pilot the *Guidelines* and demonstrate support for the approach.

**Figure:**
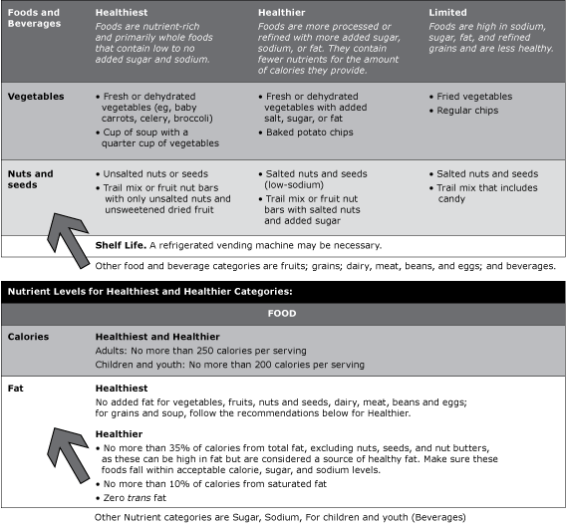
­Food and beverages in the categories of “limited,” “healthier,” and “healthiest” and nutrient levels for each category.

PHSKC staff members described the rationale for and an overview of the *Guidelines* and displayed sample items from each food category at the April 2011 KCBOH meeting. Seven stakeholders testified during the public hearing portion of the meeting. KCBOH then voted unanimously to adopt the *Guidelines* with the intention that local jurisdictions and organizations across the county use them to improve their vending machine policies and environments.

### Political stream

King County is a solidly Democratic county. Residents are favorably disposed to an activist role for government in promoting health (19). An increasing number of local efforts focus on improving access to healthy food.

KCBOH also has a history of using policy to address public health issues, including *trans* fat in restaurant food, menu labeling, healthy community planning, and tobacco use. Several KCBOH members have been active in local food system and policy development. KCBOH members generally expressed strong support for the *Guidelines*, citing a need for more healthy selections, a responsibility to make evidence-based recommendations, and an appreciation for approaches that encouraged healthy behaviors rather than banning unhealthy options. One KCBOH member said, “It’s about choices . . . . It’s all things in moderation and in healthy amounts, but we’re not out to ban Snickers bars.”

During testimony, stakeholders expressed a range of opinions about the *Guidelines* proposal (Table 2). Proponents (2 “early adopter” organizations and an obesity prevention advocacy group) described a need to increase the number of healthy vending machine options and an appreciation for the *Guidelines* as a resource to guide policy development. Vending machine companies ranged from cautiously supportive to opposed to the proposal; vendors expressed concern about their bottom lines, objected to price differentials between healthy and unhealthy foods, and anticipated a lack of demand for and availability of healthy products. One indicated that the company could “see the writing on the wall” and had started to “gear up” for the shift to healthier vending machine items. Washington’s Department of Services for the Blind (DSB) expressed concerns about anticipated profit decline. (DSB has the right of first refusal to contract for vending machines in government buildings, according to state and federal law; it uses vending machine profits to support its programs.) A KCBOH member reported that beverage industry representatives met with his staff to express similar objections.

**Table 2:** Arguments for and Against the Proposed Guidelines by Stakeholder Groups During April 2011 King County Board of Health Meeting

Arguments Made	Stakeholders					Sample Quotes
	KCBOH	VC	LJOs	HA	DSB	
**In support, or acknowledged**						
**Rationale and context**						
Health concerns (eg, obesity rates)	x	x		x		“We are seeing a shift in norms. We’re seeing a demand for a greater diversity of products in our food.” HA
Need/demand for healthy options	x	x	x	x		
Prevalence of out-of-home eating	x					
Government should model healthy environments	x			x		
**Potential outcomes**						
Increased healthy choices available	x		x	x		“When you provide a greater diversity of products, you see a shift in demand, ultimately a shift in supply.” HA
Increased demand for, supply of healthy products	x			x		
**Policy approach and implementation**						
Voluntary, not mandated	x					“These proposed Guidelines will serve as a timely and valuable tool for our agency to identify healthy vending options and to ultimately implement healthy vending practices and policies successfully.” LJO
Innovative (eg, emphasizes whole foods over nutrients alone)	x					
Evidence-based	x					
Allows for institutional flexibility	x					
Supports LJOs that want healthier vending, provides goals	x		x			
Possible to implement with limited revenue loss, few complaints		x^a^	x	x	x^a^	
**In opposition, or concerns expressed**						
**Feasibility**						
Insufficient availability of healthy items, refrigerated machines		x^a^			x^a^	“It has to be an educational process and not just putting healthy things in the machine.” VC
References specific products, but markets will change		x				
**Potential outcomes**						
Loss of revenue, negatively impacting blind services and vending viability		x			x	“There are well documented cases of how our sales drop when we go beyond a certain point of . . . healthy choices.” DSB
**Policy approach**						
Feels extreme, like a mandate		x			x	“There are very few machines in King County that are refrigerated, so you can’t put apples or carrots or fresh-made sandwiches in them.” VC
Different demographics warrant different approaches		x				
Will restrict “class of trade” (vending, but not stores)		x				
Education processes are key to process		x			x	

Abbreviations: DSB, Washington State Department of Services for the Blind; HA, health advocates; LJOs, local jurisdictions and organizations; KCBOH, King County Board of Health, VC, vending companies.

a Some stakeholders discussed thresholds whereby vending machines that had 30% to 50% healthier options would be likely to result in revenue loss, whereas vending machines with a lower percentage of healthy items might result in limited revenue loss.

The *Guidelines’* recommendation to move toward 100% healthy items in machines was the most contentious point. Both proponents and opponents cited evidence of sales declines after introducing more than 30% healthy items, but proponents emphasized that sales recovered. One KCBOH member suggested removing the targeted percentages of healthy foods, but others emphasized that the *Guidelines* were not legally binding and were based on the best science. One member said, “Our role as a board of health is really to tell citizens, ‘What do the facts say?’ We chose guidelines so we could work with people and make it happen. . . . I think we need to challenge ourselves and the industry.” KCBOH did not remove the targets.

One PHSKC staff member later summed up the advantages and disadvantages of the nonregulatory guidelines approach by saying, “It doesn’t have the force of law. We are able to do an enormous amount in terms of who the target audience is and where we set the bar for the best practice, but it also means that carrying this through to implementation . . . is going to be different for each organization and in some cases, it is going to be incredibly time-intensive.”

### Policy window

Kingdon theorizes that a policy window “opens because of a change in the political stream . . . [or] because a new problem captures the attention of governmental officials and those close to them” (11, p. 168). KCBOH’s new Guidelines and Recommendations policy category provided an opening as obesity prevention and healthy food access were gaining attention and support. The *Guidelines* provided a feasible alternative to regulations that addressed the problem of unhealthy food away from home. PHSKC staff described this concept as a “window of opportunity which we anticipate will eventually close because [KCBOH’s] attention will change to something else.”

### *Guidelines’* initial impact

#### Reach

Interviewees described measuring the reach of the *Guidelines* as a challenge, particularly for local jurisdictions and organizations with decentralized vending but noted that the potential reach was high. The networks to which KCBOH and PHSKC belonged (eg, jurisdictions represented by elected official KCBOH members, PHSKC partners) were seen as assets. Two PHSKC partners and 2 jurisdictions represented by KCBOH members used the *Guidelines* in the first year. Another KCBOH member said, however, “As [an elected official], I guarantee you, I would not go in and tell [my constituents] what vending machines they could have. I need to have an advocate from within.”

Adoption of vending machine policies by the first 4 local jurisdictions and organizations using the *Guidelines* affected approximately 345 vending machines. The total number of people reached is unknown; however, in one housing organization, approximately 460 employees and residents of 3,200 units had access to its 83 machines (20).

#### Effectiveness

Effectiveness is hard to measure because of a lack of accessible vending machine sales data. Interviewees indicated effectiveness of the *Guidelines* could be enhanced by outreach and technical assistance to promote and support use of the *Guidelines* as well as complementary behavioral change interventions.

#### Adoption

The *Guidelines* were incorporated into policy in several ways during the first year, including revised vending machine contracting requirements for 2 local jurisdictions and organizations and a county council motion for executive adoption of nutritional standards for vending machines (Table 3). Other local jurisdictions and organizations spent the first year considering policy options, including centralizing vending processes to make use of the *Guidelines* easier. Adopting organizations demonstrated strong fidelity to the nutritional guidelines and food categories, though several only required 50% healthy vending.

**Table 3:** Healthy Vending Machine Policy Form, Fidelity, and Development Highlights of Jurisdictions and Organizations That Used the King County Health Vending Guidelines, 2011–2012

Jurisdictions and Organizations	Form of Policy	Fidelity to Guidelines	Policy Development Highlights
City parks and recreation department, early adopter	Revised vending contracts to be used agency-wide	100% healthy and healthiest items with minor adaptations to *Guidelines* (eg, allows diet soda)	Prior experience with healthy vending
			Mission aligned with healthy eating, active living; very supportive leadership
			Placed strong emphasis on education and organizational culture shift
Nonprofit public housing agency, early adopter	Issued a RFP for a vending contractor to provide healthy vending throughout the organization, resulting in a contract with a new company	A minimum of 50% healthy items for all of residential and administrative vending machines^a^	Supportive leadership
			Recipient of a grant with goals pertaining to healthy eating and active living
			Residents requested healthy vending
			Convened a vending committee; conducted taste tests and price surveys; developed education materials
			Prior small vending company did not have inventory that met criteria
			Planned to increase prices in advance to limit the association of cost increases with healthier selection
			No capacity to monitor or assess contract compliance
City, effort led by KCBOH member	In 2013, passed an ordinance requiring healthy items in vending machines on city property, complementary education and labeling, and an evaluation after the first year^a^	Ordinance required 50% of items in machines to meet healthier and healthiest criteria; *Guidelines* were included as an attachment to the policy^a^	Lack of centralized contracting mechanism, and many contracts
			A staff workgroup assessed current vending and considered approaches, spoke with vending companies and beverage industry representatives
			Report submitted to City Council after first year of implementation will make recommendations for next steps
County, effort led by KCBOH member	County Council adopted a 2011 motion calling on the County Executive to adopt nutritional standards for vending machines (no standards developed by time of interview)	Motion requested standards of 50% healthiest and 25% healthier items in machines, and implement pricing and marketing strategies	Began offering 20% to 30% healthy items in some machines in 2005; sales declined initially, then improved with educational and pricing strategies; half of healthy items under the prior criteria were found to fit in the *Guidelines’* limited category.
			County Executive options under consideration at time of interview: 1) fill DSB machines with items that meet *Guidelines*; 2) replace DSB machines with healthy “kiosks”; 3) request a waiver to manage DSB machines; 4) remove machines.
			Three years later, option 2 has been rejected; contract with prior vendor continues, and additional healthy items have been added to the machines.^a^
City (out of state), learned of *Guidelines* from KCBOH member	Citywide 50% healthy vending per *Guidelines* with accompanying education	Adopted exact language of *Guidelines* as guidance for city departments; fidelity by departments to *Guidelines* not determined at time of interview	Staff had prior healthy vending experience
			Lukewarm support for *Guidelines* from political leadership
			Conducted a vending assessment
			Stakeholder pushback led to the city’s issuing an administrative order without accompanying education, charging city departments to implement their own contracts

Abbreviations: DSB, Department of Services for the Blind; Guidelines, King County Healthy Vending Guidelines; KCBOH, King County Board of Health; RFP, request for proposal.

a Details describe decisions made more than 1 year after *Guidelines* were adopted by KCBOH.

Local jurisdictions and organizations pursued vending machine policy change adoption because it seemed feasible (eg, “an area over which they could have some influence,” “an easy win”) but also reported that adoption was more complicated than anticipated. Challenges included the time and resources required, pushback from employees, and the perception that healthy vending restricts choice. The beverage industry and vending machine companies lobbied larger jurisdictions to discourage them from adopting 100% healthy items in vending machines or price differentials, requesting subsidies (unsuccessfully) to offset the expected sales reduction and losses due to expired products, and negotiating higher percentages of revenue that the companies would receive from the machines. PHSKC staff supported use of the *Guidelines* through presentations, development of a tool kit, consulting on contracting language, and identifying sources of products that met guidelines (21).

After approximately 2 years, 2 additional local jurisdictions and organizations adopted policies, including a city ordinance, requiring 50% healthy vending in all machines. A baseline evaluation of the ordinance reported that less than 10% of the city’s machines met the *Guidelines* initially (Perez J. Process evaluation report: City of Seattle implementation of King County Healthy Vending Guidelines [unpublished student report]. Seattle, Washington: Public Health Seattle King County; 2013). A local children’s hospital, community center, and low-income housing nonprofit also began changing their vending machine policies based on the *Guidelines* (20,22).

## Discussion

This case illustrates an innovative LBOH policy approach to promote healthy food access when neither legislation nor regulation is feasible or desirable. The *Guidelines’* format, along with recognized health concerns and an amenable political landscape, created a policy window for KCBOH to extend its influence in promoting evidence-based practice and affect the nutritional quality of vending machine products. The *Guidelines* catalyzed and supported several local jurisdictions and organizations in developing or strengthening vending machine policies with fidelity to the *Guidelines*, and thus to national dietary recommendations. Although there are no data to measure longer-term outcomes in this case, improvements to the nutritional quality of vending machine products have altered consumer behavior in the past (23).

Although most LBOH policy making takes the form of regulation, regulations have limitations, including politicization and conflicts with or preemption by other laws (3). By using the *Guidelines*, KCBOH and PHSKC probably avoided some “nanny-state” concerns associated with other policy approaches (24). However, even the *Guidelines* produced some pushback from stakeholders.

This qualitative study allowed for in-depth examination of the case, but the findings pertain to organizations with unique contexts and cannot necessarily be generalized to others. In recollecting events and reactions, or describing politically sensitive situations, some interviewees may have omitted important details. No sales or implementation cost data were collected. Also, vending machine company representatives declined to participate.

As this study focused on the development and preliminary uptake of the *Guidelines*, future research could examine implementation of vending machine policies based on such guidelines as well as longer-term use of or changes made to nonregulatory tools based on evolving perceptions of the problem, tool, or political realities. Furthermore, studies could assess vending machine sales in particular jurisdictions over time and consider the health implications and facilitators and barriers to those changes.

Experts have called for policy approaches to prevent obesity and chronic disease by improving access to healthy food and addressing other determinants. LBOHs and communities could benefit from KCBOH’s experience in using nonregulatory evidence-based guidelines as one policy tool toward this end.
